# A novel strategy to characterize the pattern of β-lactam antibiotic-induced drug resistance in *Acinetobacter baumannii*

**DOI:** 10.1038/s41598-023-36475-9

**Published:** 2023-06-06

**Authors:** Trae Hillyer, Bogdan M. Benin, Chuanqi Sun, Noah Aguirre, Belinda Willard, Yuk Yin Sham, Woo Shik Shin

**Affiliations:** 1 Department of Pharmaceutical Sciences, Northeast Ohio Medical University, Rootstown, OH USA; 2 Department of Neurology, University of California, Los Angeles, CA USA; 3 Proteomics and Metabolomics Core, Lerner Research Institute, Cleveland Clinic, Cleveland, OH USA; 4 Department of Integrative Biology and Physiology, University of Minnesota, Minneapolis, MN USA

**Keywords:** Microbiology, Medical research, Pathogenesis

## Abstract

Carbapenem-resistant *Acinetobacter baumannii* (CRAb) is an urgent public health threat, according to the CDC. This pathogen has few treatment options and causes severe nosocomial infections with > 50% fatality rate. Although previous studies have examined the proteome of CRAb, there have been no focused analyses of dynamic changes to β-lactamase expression that may occur due to drug exposure. Here, we present our initial proteomic study of variation in β-lactamase expression that occurs in CRAb with different β-lactam antibiotics. Briefly, drug resistance to Ab (ATCC 19606) was induced by the administration of various classes of β-lactam antibiotics, and the cell-free supernatant was isolated, concentrated, separated by SDS-PAGE, digested with trypsin, and identified by label-free LC–MS-based quantitative proteomics. Thirteen proteins were identified and evaluated using a 1789 sequence database of Ab β-lactamases from UniProt, the majority of which were Class C β-lactamases (≥ 80%). Importantly, different antibiotics, even those of the same class (e.g. penicillin and amoxicillin), induced non-equivalent responses comprising various isoforms of Class C and D serine-β-lactamases, resulting in unique resistomes. These results open the door to a new approach of analyzing and studying the problem of multi-drug resistance in bacteria that rely strongly on β-lactamase expression.

## Introduction

*Acinetobacter baumannii* (Ab), an aerobic Gram-negative coccobacillus, is one of the ESKAPE pathogens and is currently classified as an urgent threat to public health by the CDC^1^. This classification is due to the severity and high mortality (in some cases greater than 50%) of carbapenem-resistant Ab (CRAb) infections^2–8^. Additionally, these infections are generally nosocomial and frequently occur in the intensive care unit (ICU; accounting in some cases for up to 31% of ICU infections around the world) where patients are already more sensitive^2,9–12^.

To develop new therapeutic strategies to combat these pathogens, a deeper understanding of their resistance mechanisms is required. Typically, bacteria utilize a combination of target modification, influx/efflux regulation, metabolic changes, and drug deactivation through the expression of β-lactamases, but the relative contribution of these multiple strategies varies from pathogen to pathogen^7,13^. For example, although Ab and CRAb can express modified penicillin binding protein (PBP), it is not generally regarded as the primary mechanism of resistance unlike methicillin resistant *Staphylococcus aureus*^14^. Regarding CRAb specifically, early studies presented opposing views on the relative importance of PBP modifications and regulation, with more recent reviews suggesting that carbapenemase production tends to be the most significant method of resistance for CRAb^14–16^. Carbapenemases, briefly, are β-lactamases that can hydrolyze carbapenems in addition to other β-lactam antibiotics. Examples of these are found in class A and D serine β-lactamases and class B metallo-β-lactamases, with the class D OXA-type serine β-lactamase being regularly detected in CRAb^17–19^. Previous studies have also reported that a variety of such OXA-type β-lactamases can be found in CRAb^19^; however, there has been limited work done on attempting to selectively characterize the entire set of β-lactamases in a single strain and compare these with resistant mutants^20–23^.

Cataloguing and analyzing the collection of these enzymes may therefore be a critical step in the development of novel combination therapies in which β-lactamase inhibitors are combined with β-lactam antibiotics. One recent report even demonstrated a novel combination of β-lactamase inhibitors being used in the successful treatment of a patient suffering from an XDR Ab infection. However, since inhibitors themselves are not effective against all classes, there exists the possibility that future β-lactamase mutations can render inhibitors ineffective^24–26^. The difficulty in this lies in the fact that drug-resistant bacteria can carry multiple copies of a β-lactamase gene, which do not need to be simultaneously expressed (or at least not to equal extent) in order to maintain efficient cell growth. Therefore, bacteria may express unique collections of β-lactamases, resulting in specific resistomes that are not only antibiotic-class (e.g. β-lactams) but also molecule dependent. Furthermore, it is unknown to which extent these resistomes retain a “memory” of the previous antibiotic exposure or as to how quickly they can adapt to new environmental stressors.

To understand antibiotic resistance in bacteria and what factors may influence it, many studies have utilized various “-omic” approaches to characterize the genetic (genomic), transcriptional (transcriptomic), metabolic (metabolomics), and translational (proteomic) changes that may occur in bacteria as a result of drug administration. Among these omics, proteomics provides the most direct information regarding the bacterial response to external stimuli such as antibiotic usage. Therefore, many recent studies report the proteomic profiles or proteomes of drug-resistant clinical isolates as well as bacteria with drug resistance which was induced in the laboratory^20,27–30^. Typically, the whole proteome is measured and to show differential expression of numerous proteins in various drug-resistant bacteria, including those related to metabolism, reactive oxygen species management, drug targets, DNA/RNA modification, etc. In the case of the proteomes of antibiotic-resistant Ab strains, researchers observed that β-lactamase expression was generally upregulated^20,27,31,32^. However, though a small number of studies have observed a correlation between various antibiotics and total protein expression in Ab, there has been no systematic investigation of specific antibiotic exposure (same or different classes) on bacteria and their specific enzymatic responses. In support of such a study, two recent reports specifically identified previous antibiotic usage and β-lactamase inhibitor exposure as risk factors for drug-resistant Gram-negative infections^5,33^. These studies and reports together suggest that genes, proteins, drug structures, and their specific functions are all interconnected to develop drug resistance. Therefore, we could hypothesize that the structural differences between various β-lactam antibiotics may be important for different bacterial resistance responses in the form of altered β-lactamase expression patterns.

Herein, we a present the targeted LC–MS-based quantitative proteomic study of the β-lactamase expression of Ab (ATCC 19606; Ab19606) in response to exposure to various β-lactam antibiotics. This was accomplished through the separation of the cell-free supernatant from the bacterial growth medium using SDS-PAGE, followed by LC–MS–MS analysis of the protein mixture.

## Results

### Antibiotic exposure and characterization of β-lactam resistance

**Figure 1:**
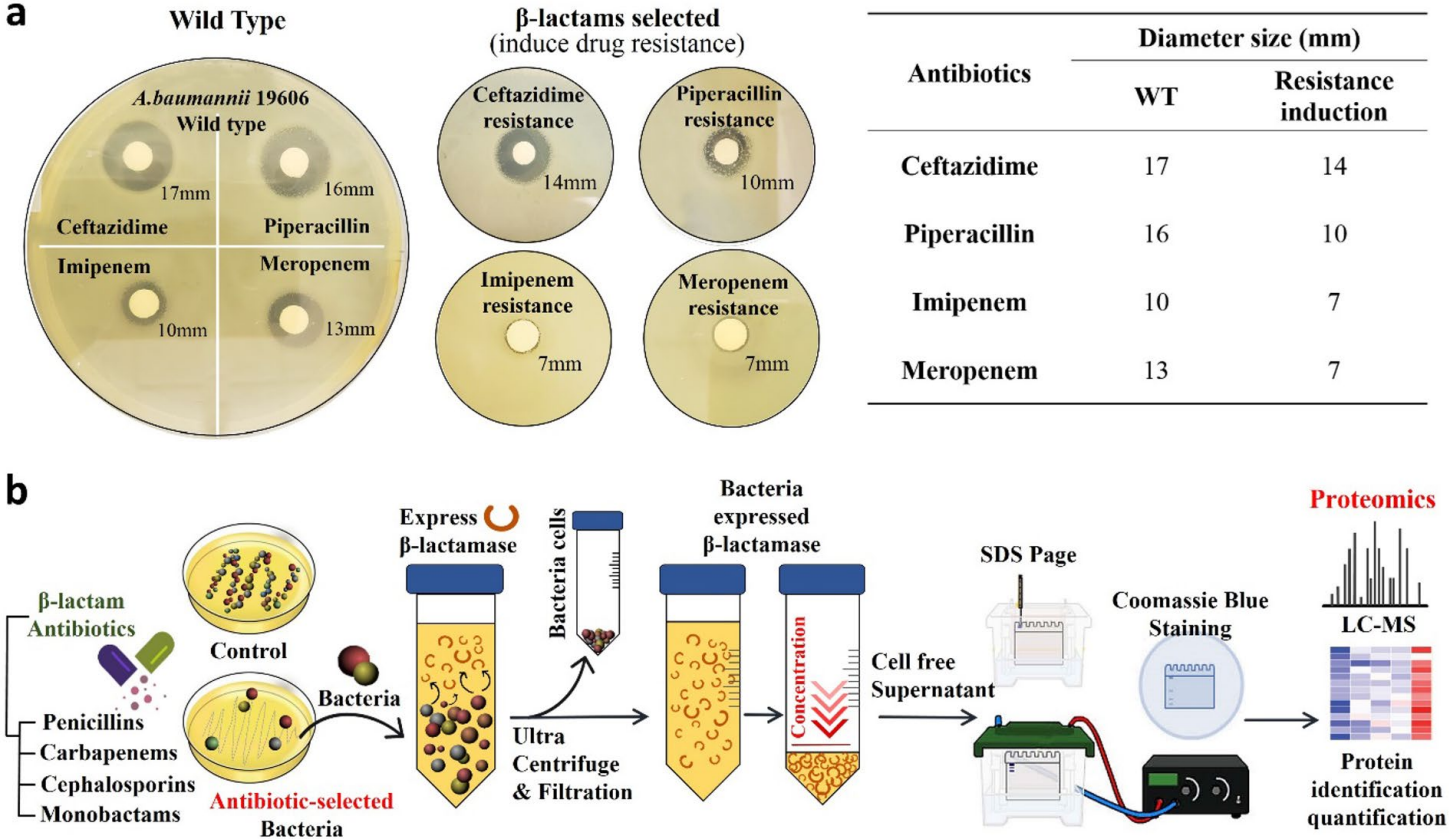
**(a)** Disk diffusion assay was performed via wild-type and β-lactams-selected Ab19606 strain to confirm antibiotic treatment-induced resistance. The induced resistance was determined by measuring the size of the diameter, and all the resistance was confirmed through triplicate repetitions. (**b**) Experimental scheme of β-lactam antibiotic selection and sample separation/preparation for proteomics approach.

To determine and characterize β-lactam resistance in Ab19606, which has been wildly used as a control strain in studies involving antibiotic resistance, was cultured in nutrient broth media with four different classes of β-lactam antibiotics (10 µg/mL). To confirm that resistance was induced by repeated β-lactam exposure, disk diffusion assays were conducted on Mueller–Hinton (MH) agar (Fig. 1a). Colonies were observed grown after 24 h of incubation at 37 °C. In comparison to the wild-type drug-sensitive ATCC 19606, the inhibitory regions were reduced for all antibiotics against the drug-resistant strains: ceftazidime (cephalosporin) and piperacillin (penicillin) were reduced by 5 mm and 6 mm, respectively, and imipenem (carbapenem) and meropenem (carbapenem) were reduced by 7 mm and 5 mm, respectively. This confirmed that Ab19606 could generate significant β-lactam resistance to the exposed antibiotics.

To further evaluate the mechanism of resistance occurring in these organisms, Ab19606 was grown in nutrient broth and plated on agar plates containing sub-inhibitory concentrations of various beta-lactam and non-beta-lactam antibiotics. Colonies were selected and grown in one-liter flasks of nutrient broth with a 5 μM concentration of the corresponding antibiotic. Once the culture reached stationary phase, the cells were centrifuged out, and the supernatant was concentrated using Ultra-15 centrifugal filter units (Fig. 1b).

### Apparent kinetic parameter determination

To verify the presence of β-lactamase enzymes from the β-lactam-selected Ab19606 strains, nitrocefin, a chromogenic cephalosporin, was used as a colorimetric indicator^34,35^. Initially, this cell-free supernatant was compared with the cell lysate to ensure that β-lactamases were present (Supplementary Fig. S1). Typically, it is considered that only Gram-positive bacteria excrete β-lactamases into the extracellular space^36^. However, several publications have demonstrated that Gram-negative bacteria, such as Ab, are also able to do so^37–42^. This is qualitatively supported by our experiment demonstrating the ability of the cell-free supernatant to also hydrolyze nitrocefin. For all concentrated, cell-free supernatant samples, enzymatic activity could be detected and biochemical activity parameters (e.g. *K*_m_, *k*_cat_) could be obtained by varying the nitrocefin concentration from 0.01 to 75 μM (Table 1, Supplementary Fig. S2). A sample of purified TEM-1 β-lactamase was used as a positive kinetics control. The apparent *K*_m_ and *k*_cat_ results are comparable with TEM-1 control kinetic parameters, showing a reasonable range for *kcat*/*Km*^43^. These results suggest that not only were β-lactamase enzymes present within the concentrated cell-free supernatant, but they were also within a suitable concentration range and that further characterization could proceed.

**Table 1:** β-lactamase activity of antibiotic selected Ab19606-free supernatant solutions as compared to TEM-1 using nitrocefin as a colorimetric substrate.

Enzyme & selection	Antibiotic class	${\mathrm{K}}_{\mathrm{m}}^{\mathrm{app}}$ (μM)	${\mathrm{k}}_{\mathrm{cat}}^{\mathrm{app}}$ (s^−1^)	${\mathrm{k}}_{\mathrm{cat}}^{\mathrm{app}}/{\mathrm{K}}_{\mathrm{m}}^{\mathrm{app}}$(μM^−1^ s^−1^)
TEM-1 (control)	–	38.7 ± 4.1	153.2 ± 21.3	3.95
Penicillin G	Penicillins	14.1 ± 3.2	170.3 ± 48.6	12.07
Meropenem	Carbapenems	15.5 ± 4.7	213.3 ± 12.6	13.76
Faropenam	Penems	27.2 ± 6.6	142.7 ± 17.5	5.24
Aztreonam	Monobactams	12.8 ± 2.2	247.1 ± 13.2	19.3
Piperacillin	Penicillins	11.2 ± 2.9	104.6 ± 10.7	9.33
Imipenem	Carbapenems	24.5 ± 8.1	121.7 ± 19.8	4.96
Ceftazidime	Cephalosporins	21.7 ± 9.3	151.2 ± 14.1	6.96
Amoxicillin	Penicillins	16.8 ± 6.5	264.4 ± 21.7	15.73

### Separation and confirmation of β-lactamases by SDS gel page and Proteomics study

**Figure 2:**
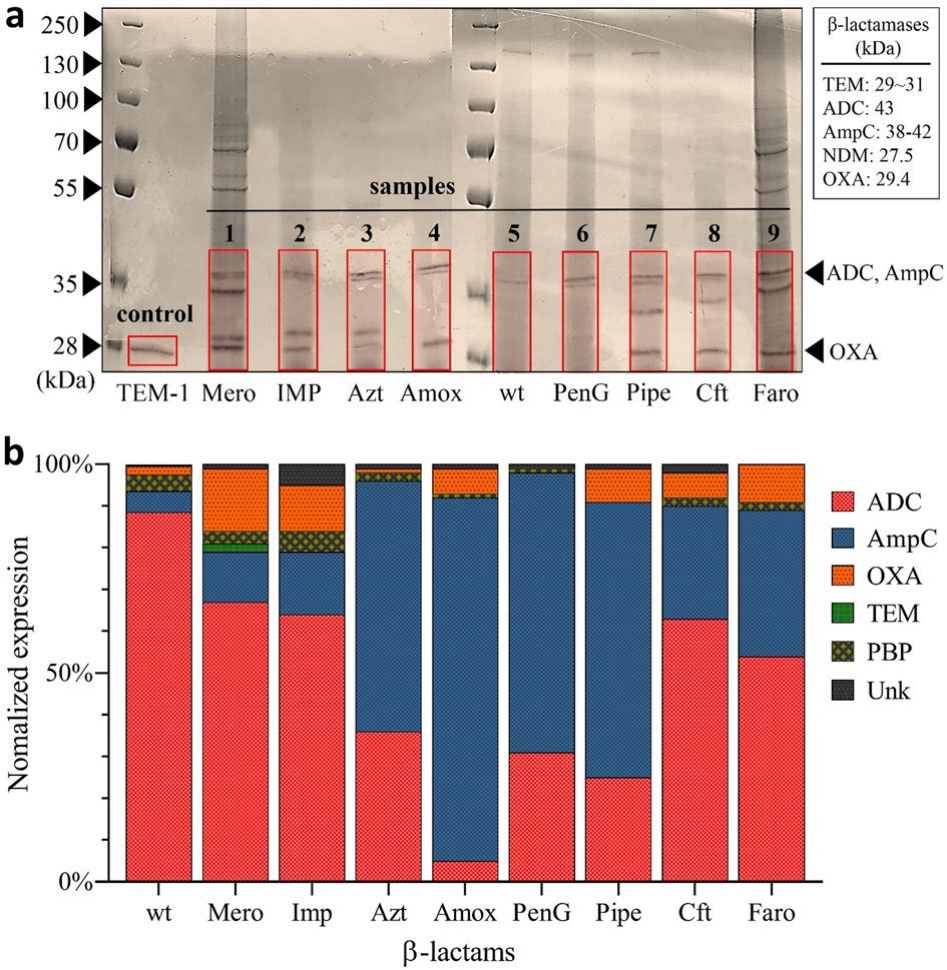
**(a)** SDS-PAGE and Coomassie blue staining of cell-free supernatant samples. Purified TEM-1 β-lactamase was used as a control. The possible area of gel containing all classes of β-lactamase proteins (red square) was cut for further proteomics analysis. (**b)** Relative proportions of β-lactamases expressed after antibiotic exposure. ADC and AmpC are type C β-lactamases; OXA is type D; TEM is type A; PBP is the target of β-lactam antibiotics but has structural similarities and a similar molecular weight.

To visualize and separate the β-lactamases expressed by Ab19606, in preparation for LC–MS, we performed an SDS gel separation of the highly concentrated supernatant samples that came from Ab19606 samples after 72 h exposure to 5 μM antibiotics (Fig. 2a). A purified class A β-lactamase (TEM-1, 29 kDa/line 1) was used as a positive control line. Following SDS-PAGE, d-lactamase proteins were readily visible after both Coomassie blue staining. Several lanes containing supernatants collected from various β-lactam exposed Ab19606 samples (samples 1–9) exhibited a distinct band at a location corresponding to a size between 27.5 and 42 kDa, corresponding to the expression of β-lactamases. Interestingly, the intensity of the bands was variable and the separated protein gel bands showed different protein patterns and expression levels depending on the antibiotic used to induce resistance.

After the successful separation of proteins using SDS gel electrophoresis, sections of the gel containing the β-lactamase proteins (red regions in Fig. 2a) were cut for proteomics analysis. The LC–MS/MS based proteomics experiments were performed using a label-free proteomics method (MaxLFQ) for the identification of the β-lactamase isoforms expressed by the drug resistant colonies. More specifically, the sample preparation was performed by tryptic digestion and the digested samples were analyzed by high-resolution liquid chromatography-mass spectrometry (LC–MS/MS). The identified peptides were then analyzed and evaluated through Mascot, Proteome Discoverer, and MSFragger using a FASTA file comprising Ab19606 β-lactamase sequence database.

Across all samples, various sequences were identified that match with, with at least 2 unique peptide sequences, to various β-lactamase isoforms (Table 2). The relative abundance of these isoforms was then compared by normalizing the label-free intensity to the total measured intensity for the sample. Various proteins, which belong to a larger class were then grouped together, e.g., OXA-51 and OXA-66 are combined into one OXA group (Fig. 2b). Although various non-β-lactamase proteins were also identified from the proteomics analysis, the most prominent protein types were ampicillinase C (AmpC), *Acinetobacter-*derived AmpC (ADC), and oxacillinase (OXA), which belong to both class C and D enzymes (Fig. 2b, Supplementary Fig. S3). Importantly, the expression of these enzymes by Ab19606 agrees with the presence of both *bla*_AmpC_ and *bla*_OXA_ genes catalogued by the ATCC.^44^ Interestingly, the relative amounts of these three types of β-lactamase proteins were observed to be significantly different depending on the antibiotic to which Ab19606 was exposed. This data suggests that the expression of β-lactamases may be influenced by the specific antibiotic treatment, especially in cases where Ab19606 was exposed to antibiotic concentrations above its MIC. This concept is further supported by the observation that these active resistance profiles are also different for antibiotics of the same class. For example, penicillin G(penG), amoxicillin (amox), and piperacillin (pipe) are all penicillin class β-lactam antibiotics, yet they produce quite different responses (Fig. 2b, Supplementary Fig. S3).

**Table 2:** Obtained major peptide sequence by LS–MS mass spectrometry.

Sequence	Peptide mass	Mapped gene	Protein ID
AAYAVLDAIKK	1161.6	AmpC	A0A0R4J6T7
KKAVNRSTIFE	1291.7	AmpC	A0A0R4J6T7
DWQPKNPIGEYR	1501.7	AmpC	A0A009PJF4
FIYANLNPQKYPADIQR	2050.1	AmpC	A0A009PJF4
TQMQNYDFGYNQENQPIR	2244.9	AmpC	A7Y413
ASAIPVYQDLAR	1302.6	OXA	A0A009HC83
ASTEYVPASTFK	1299.6	OXA	A0A009HC83
ATTTEVFKWDGQKR	1665.8	OXA	A0A009HC83
GIPSSVRK	842.4	OXA	A0A009HC83
IGLELMSNEVKR	1387.7	OXA	A0A009HC83
IKNLFNEAHTTGVLVIQQGQTQQSYGNDLAR	3442.7	OXA	A0A009HC83
ITPQQEAQFAYK	1422.7	OXA	A0A009HC83
KGIPSSVR	842.4	OXA	A0A009HC83
LFPEWEK	947.4	OXA	A0A009HC83
MLNALIGLEHHK	1374.7	OXA	A0A009HC83
NMTLGDAMK	979.4	OXA	A0A009HC83
RIGLELMSNEVKR	1543.8	OXA	A0A009HC83
VGYGNADIGTQVDNFWLVGPLK	2362.1	OXA	A0A009HC83
TFFKDWKPKNPIG	1576.8	ADC	A0A5C1K4D3
AVGYNQENQPIRVNPG	1754.8	ADC	A0A5C1K4D3
STLPDMLSFIHANLNPQKYPTDIQR	2898.4	ADC	A0A5C1K4D3
GSVSKLFNATAGGYA	1441.7	ADC	A0A5C1K4D3
TQMQNYAVGYNQENQPIR	2152.9	ADC	A0A5C1K4D3
QMQNYAFGYNQENQP	1830.7	ADC	A0A5C0PFX8
KTGTTTGFGTYVVFI	1590.8	ADC	A0A5C0PFX8

## Discussion

Antibiotics have given humanity a successful edge against pathogens over the past half-century. However, mutations and natural selection, combined with fast generation times and enormous population sizes, are now giving pathogens a decisive advantage^45–48^. To regain the upper hand, it is important to better understand the relationship between antibiotics structure and function and how pathogens can systematically evolve to subvert them so that new treatment strategies may be designed.

Bacteria have steadily developed resistance to many of the classes of antimicrobial agents currently in use. Some bacteria, such as Ab, have a propensity to develop high levels of drug-resistance, thus being classified as extensively drug resistant (XDR) and pan drug resistant^6,46,49,50^. Our choice of Ab19606 and β-lactam antibiotics was therefore specifically informed by the fact that CRAb is now considered a priority threat by the CDC as there are few treatments available once an infection has occurred^1,46,51^. Furthermore, carbapenem resistance is often found in strains that are considered MDR or XDR^50,52,53^. Although Ab and CRAb, like many bacteria, have multiple modes of resistance available to them, some consider that the deactivation of β-lactams through the action of β-lactamases may be the most significant mechanism^54,55^. This presents several challenges since the β-lactamases are numerous, have high similarity, easily transferrable among bacteria, and readily mutate to provide greater activity in resource-limited environments. However, we consider that these same characteristics could also provide an opportunity to fingerprint the non-specific or unintended interactions of antibiotics with bacteria that result in resistance.

**Figure 3:**
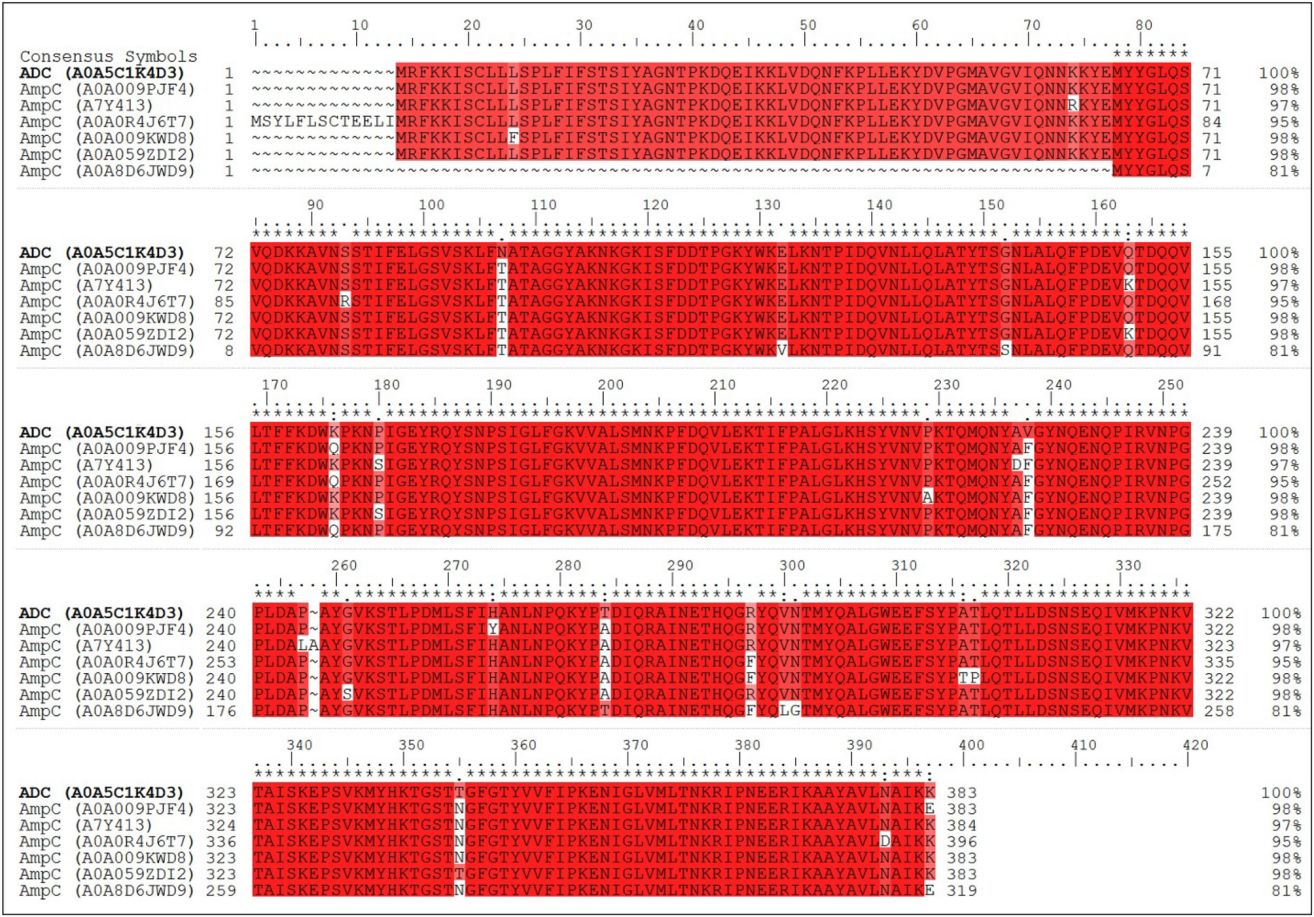
Multiple sequence comparison of identified AmpC (A0A009PJF4 to A0A8D6JWD9) and ADC (A0A5C1K4D3) isoforms. Red indicates that the residue matches the reference sequence (AmpC). The figure was generated using the program prime which is Schrodinger package.

Our findings, presented in Fig. 2 and Supplementary Fig. S3, demonstrate the variability in β-lactamase expression that can occur as a result of antibiotic exposure. Importantly, all antibiotics resulted in expression profiles that are significantly different from that of the wild-type Ab19606, which was found to predominantly express ADC. Unique sequences could identify each enzyme and were used for the MaxLFQ quantification of protein expression. We further demonstrate through side-by-side sequence comparison that ADC (A0A5C1K4D3) and AmpC (A0A009PJF4 to A0A8D6JWD9) enzymes are indeed unique isoforms (Fig. 3)^56^.

The coverage maps of these and other identified proteins are provided in the Supplementary Fig. S4. Interestingly, the class C β-lactamases, which include AmpC and ADC, had very high variability among the various Ab19606 samples, but appear to demonstrate a β-lactam sub-class dependence (Supplementary Fig. S5). More specifically, carbapenem treated samples (especially meropenem and imipenem) expressed the most AmpC (A0A009KWD8) with a large proportion of ADC. Correspondingly, samples treated with the more common penicillin-derived β-lactams such as penicillin G, amoxicillin, and piperacillin all, generally, resulted in much greater proportions of AmpC (A0A0R4J6T7). This difference could be due to an inability of Class-C β-lactamases to cleave carbapenems, while readily hydrolyzing penicillins or cephalosporins^14,17,57^. This would suggest that the bacteria treated with carbapenems are under greater stress, which leads to greater β-lactamase expression and a larger degree of mutation and the greater presence of related isoforms.

This same concept appears to extend to the variable expression of OXA and PBP proteins, even though no clear trend can be observed. The variable expression of OXA is important, however, as these Class D β-lactamases are known carbapenemases and are involved in the evolution of CRAb^17,19,52^. In our data, it appears that all carbapenem-class β-lactams did induce the expression of OXA, with meropenem and faropenem resulting in the greatest relative amount among all samples. It is unclear why imipenem did not necessarily follow this trend, or why penicillin, amoxicillin, and piperacillin all have different but lesser levels of OXA expression.

The observation of these initial differences between β-lactam classes and even between compounds of the same sub-class are promising and suggests a more complex relationship between antibiotic structure and resistance development than has been previously reported. Still, it is unclear how well these studies may correlate to in-vivo resistance generation as the concentration of compound will vary greatly in-vivo and be impacted by distribution, metabolism, and bacterial count. Furthermore, our study could not take into account the effect that polymicrobial populations may have on resistance due to gene transfer.

## Conclusion

Through the use of a label-free proteomic method in which the β-lactamases present in a cell-free supernatant solution were analyzed, it was observed that Ab19606 had produced different profiles of β-lactamases for each β-lactam antibiotic that was applied. These results suggest that the specific β-lactam, *ergo* its structure, may affect the same bacteria differently, suggesting that there exists a more complicated relationship between antibiotic structure/function and resistance generation. Future studies will investigate the possibility that this variation exists within classes, is not random, and if concentration-dependent trends may be identified. The further elucidation of such relationships would not only significantly expand our understanding of bacterial resistance mechanisms, but it could also lead to critical new tools for the design of next-generation antibiotics or combination therapies that could possibly allow for the inhibition or evasion of β-lactamase-based resistance.

## Materials and methods

### Disk diffusion assays

Ab19606 was grown in nutrient broth at 37 °C overnight. The culture was diluted to 0.5 McFarland standard (1.5 × 10^8^ CFU/mL) and 100 μL was spread onto Mueller–Hinton agar. Appropriate amounts on antibiotic were added to 6 mm disks in accordance to Clinical and Laboratory Standards Institute (CLSI). Plates were incubated at 37 °C for 18 h before zones of inhibition were determined. For subsequent exposure, bacteria were collected along the zone of inhibition of a disk, and re-cultured in nutrient broth. Cells were prepared identically, however, each disk diffusion assay plate only had antibiotic disks (3×) matching that from the disk creating the zone of inhibition the bacteria were collected from. Subsequent assays were carried out until mutations allowing for resistance to occur appeared, typically in 2–3 passages.

### Culture conditions for Ab19606 β-lactamase expression

Ab19606 was grown at 37 °C in nutrient broth overnight and diluted to 0.5 McFarland standard (1.5 × 10^8^ CFU/mL). To induce expression of β-lactamases, cultures of Ab19606 were spread on nutrient agar plates containing sub-inhibitory concentrations of antibiotic for colony isolation using the streak method and incubated at 37 °C for 24 h. Colonies were then selected and grown, with shaking, at 37 °C for 72 h in 1 L of nutrient broth with 5 μM of the same antibiotic that was used for selection.

### Supernatant collection and purification

The one-liter cultures previously described were used for analyzing the β-lactamase production induced by the antibiotic present in the media. After 72 h incubation, the media was centrifuged twice (8000×*g*, 10 min). We filtered the clarified supernatant through a 0.2 μm syringe filter to remove any remaining bacteria pathogens. The entire supernatant was then concentrated using Millipore Sigma Ultra-15 centrifugal filter units with 10 kDa cutoff (Catalog No. UFC901008).

### β-Lactamase activity and apparent kinetic assays

TEM-1 was expressed in *Escherichia coli* BL21 (DE3) with pET-TEM-1 vector, extracted by osmotic shock, and purified by Zn-chelating chromatography and gel filtration (sephacryl-100). 50 mM Tris, pH 8.0, and 150 mM NaCl were used for storage.

The purified TEM-1 and β-lactamases in the supernatant activity were determined spectrophotometrically (spectramax-M5-reader) at room temperature in 50 mM potassium phosphate buffer (pH 7.0) that contributes to enzyme stability at these volumes in a total volume of 100 µl under the conditions with nitrocefin (ε486 nm = 20,500 M^−1^ cm^−1^) as reporter substrate. Nitrocefin (0.001 to 100 μM) was freshly prepared in 50 mM potassium buffer (pH 7.0). The apparent Km and kcat values were derived from at least four independent initial velocity measurements by applying a nonlinear regression fit with the Michaelis–Menten enzyme kinetics model in GraphPad Prism 9.

### SDS gel electrophoresis and staining

Concentrated supernatant samples (7.5 μL) were mixed in 1.5 mL microcentrifuge tubes with 2 × Laemmli buffer stain, Bio-Rad (2.5 μL). The samples were heated in a water bath for 10 min at 100 °C and then centrifuged (12,000 rpm, 10 min). The proteins in antibiotic-selected bacterial pathogens supernatant were separated by SDS-PAGE 10% gradient Novex Tris–glycine resolving gel (Invitrogen, Carlsbad, CA, USA). Following electrophoresis separation at 130 V for 1 h, the gel was fixed in 50% MeOH, 10% HoAC, 40% H_2_O for 20 min. The gels were placed in a plastic tray containing an appropriate volume (100-250 mL) of staining solution (0.25% Coomassie Blue R-250) until the gel was a uniform blue color. Staining was completed when the gel was no longer visible in the dye solution. For destaining the gel, 5% MeOH, and 7.5% HoAC in 87.5% dH_2_0 were used until the background was transparent. The gels were stored in 7% HoAC.

### Proteomics analysis

For the protein digestion, the bands were cut to minimize excess polyacrylamide, divided into a number of smaller pieces. The gel pieces washed with water and dehydrated in acetonitrile. The bands were then reduced with DTT and alkylated with iodoacetamide prior to the in-gel digestion. All bands were digested in-gel using trypsin, by adding 5 μL 10 ng/μL trypsin or chymotrypsin in 50 mM ammonium bicarbonate and incubating overnight digestion at room temperature to achieve complete digestion. The peptides that were formed were extracted from the polyacrylamide in two aliquots of 30μL 50% acetonitrile with 5% formic acid. These extracts were combined and evaporated to < 10 μL in Speedvac and then resuspended in 1% acetic acid to make up a final volume of ~ 30 μL for LC–MS analysis. The LC–MS system was a Bruker TimsTof Pro2 Q-Tof mass spectrometry system operating in positive ion mode, coupled with a CaptiveSpray ion source (both from Bruker Daltonik GmbH, Bremen). The HPLC column was a Bruker 15 cm × 75 μm id C18 ReproSil AQ, 1.9 μm, 120 Å reversed-phase capillary chromatography column. One μL volumes of the extract were injected and the peptides eluted from the column by an acetonitrile/0.1% formic acid gradient at a flow rate of 0.3 μL/min were introduced into the source of the mass spectrometer on-line. The digests were analyzed using a Parallel Accumulation-Serial Fragmentation DDA method was used to select precursor ions for fragmentation with a TIMS-MS scan followed by 10 PASEF MS/MS scans. The TIMS-MS survey scan was acquired between 0.60 and 1.6 Vs/cm^2^ and 100–1700 *m/z* with a ramp time of 166 ms. The total cycle time for the PASEF scans was 1.2 s and the MS/MS experiments were performed with a collision energies between 20 eV (0.6 Vs/cm^2^) to 59 eV (1.6 Vs/cm^2^). Precursors with 2–5 charges were selected with the target value set to 20,000 a.u and intensity threshold to 2500 a.u. Precursors were dynamically excluded for 0.4 s. The data were analyzed by using all CID spectra collected in the experiment to search an *Ab* database compiled using Uniprot using the program MSFragger. The parameters for this search include a precursor mass accuracy of 20 ppm and fragment mass accuracy of 0.05 Da, fully tryptic peptides with two allowed missed cleavages, oxidized methionine and protein N-terminal acetylation as variable modifications, and carbamidomethylation as a static modification. Protein and peptide identification were validated to 1% FDR using a decoy database strategy.

### Multiple-sequence analysis

Our sequence alignment method was used for database search in a straightforward manner. The multiple sequence alignment tools in Schrodinger package ver. 2019-3 based on classic Smith-Waterman algorithm were used. The comparing sequence data base were provided by UniProt and NCBI Protein Data Bank.

## Supplementary Information

Supplementary Figures.

## Data Availability

The datasets generated during and/or analyzed during the current study are available from the corresponding author upon reasonable request. Also, the mass spectrometry proteomics data have been deposited to the ProteomeXchange Consortium via the PRIDE partner repository with the dataset identifier PXD042336.
