# Supplementary material for: A novel strategy to characterize the pattern of β-lactam antibiotic-induced drug resistance in Acinetobacter baumannii
Source: Sci Rep. 2023 Jun 6;13:9177. doi: 10.1038/s41598-023-36475-9 (PMC10244389; doi:10.1038/s41598-023-36475-9)
Supplement: Supplementary file 1 — Supplementary Figures. [file 41598_2023_36475_MOESM1_ESM.docx]

**Supplementary information**

**A novel strategy to characterize the pattern of β-lactam antibiotic-induced drug resistance in *Acinetobacter baumannii.***

Trae Hillyer^#,1^, Bogdan M. Benin^#,1^, Chuanqi Sun^2^, Noah Aguirre^1^, Belinda Willard^3^, Yuk Yin Sham^4^, Woo Shik Shin^1^*

^1^Department of Pharmaceutical Sciences, Northeast Ohio Medical University, Rootstown, OH USA

^2^Integrated Pharmaceutical Medicine Program, Northeast Ohio Medical University, Rootstown, OH USA

^3^Proteomics and Metabolomics Core, Lerner Research Institute, Cleveland Clinic, Cleveland, OH USA

^4^Department of Integrative Biology and Physiology, University of Minnesota, Minneapolis, MN USA

#Equal Contribution

*Corresponding author: Woo Shik Shin


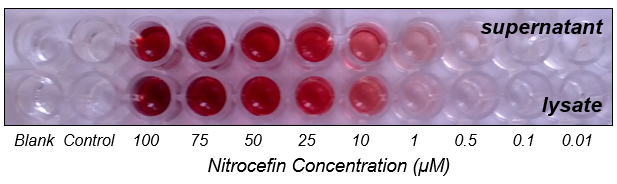


**Supplementary Figure S1**. Enzymatic activity of Ab19606 cell lysate and supernatant against Nitrocefin.


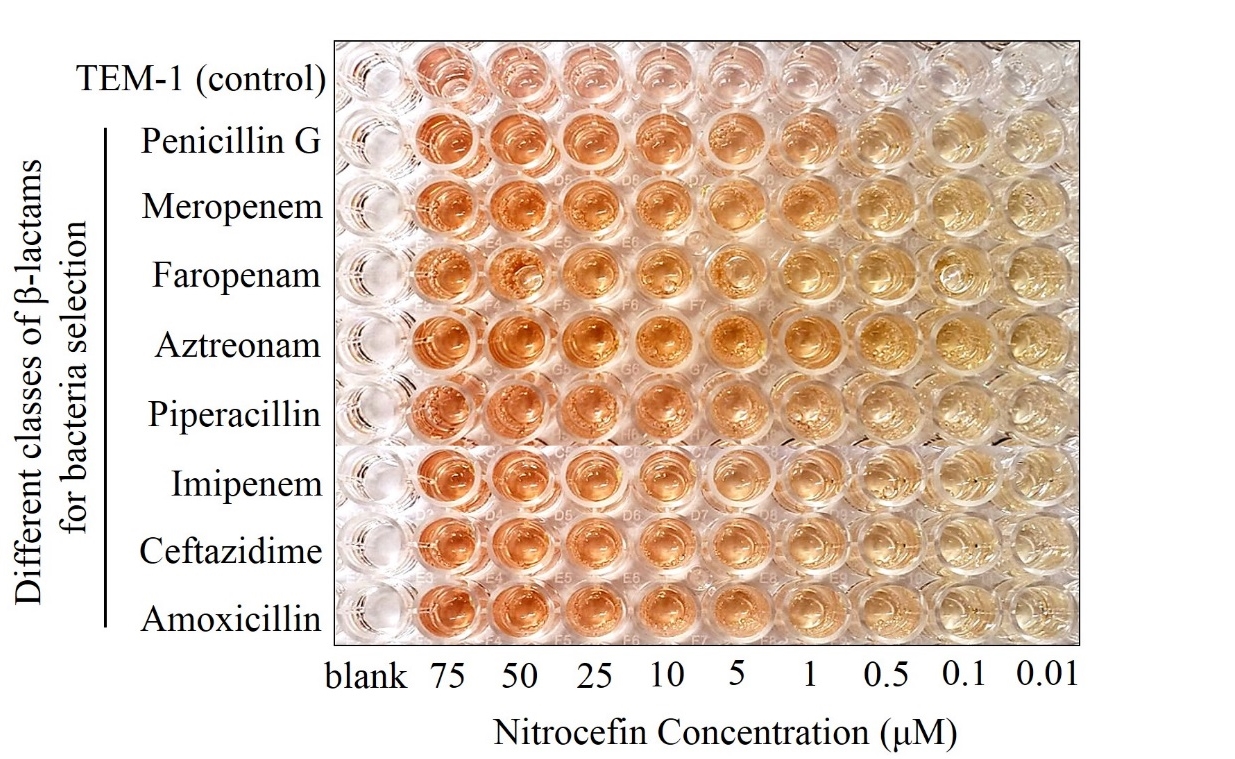


**Supplementary Figure S2.** Plate image for checking the enzymatic activity of β-lactam-selected *Acinetobacter baumannii* supernatant against nitrocefin substrate.


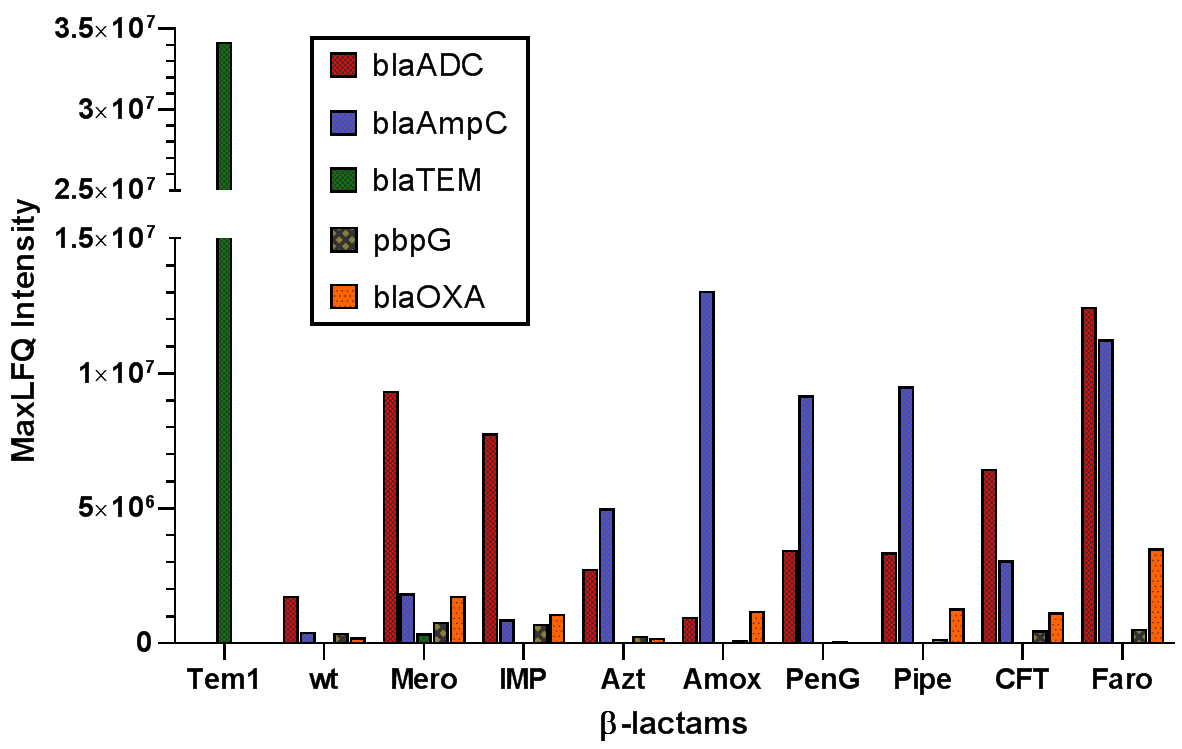


**Supplementary Figure S3.** Label free intensity (MaxLFQ) distribution of expressed β-lactamase across samples.

**Supplementary Figure S4.** Identified peptide sequences from proteomics analysis.
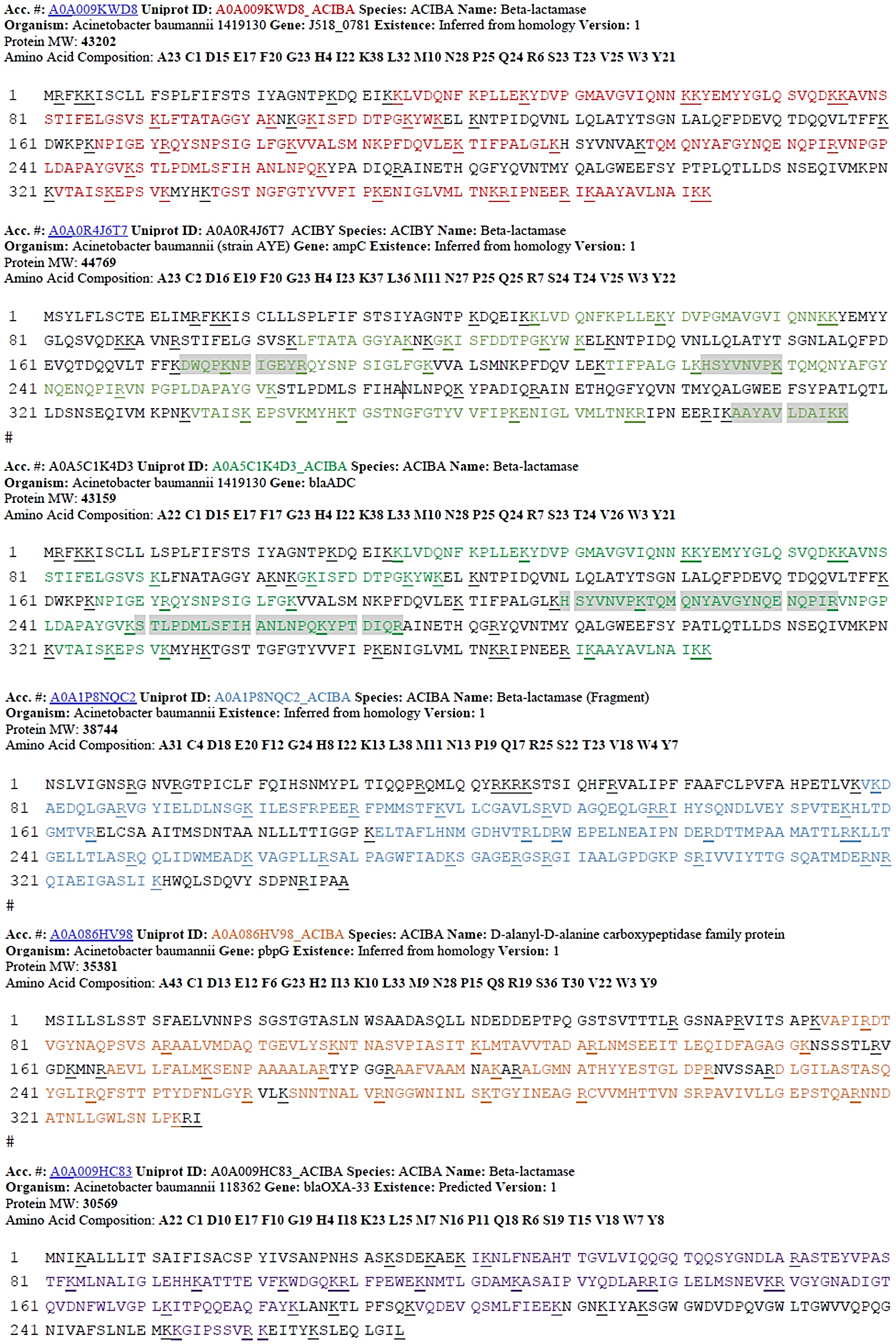


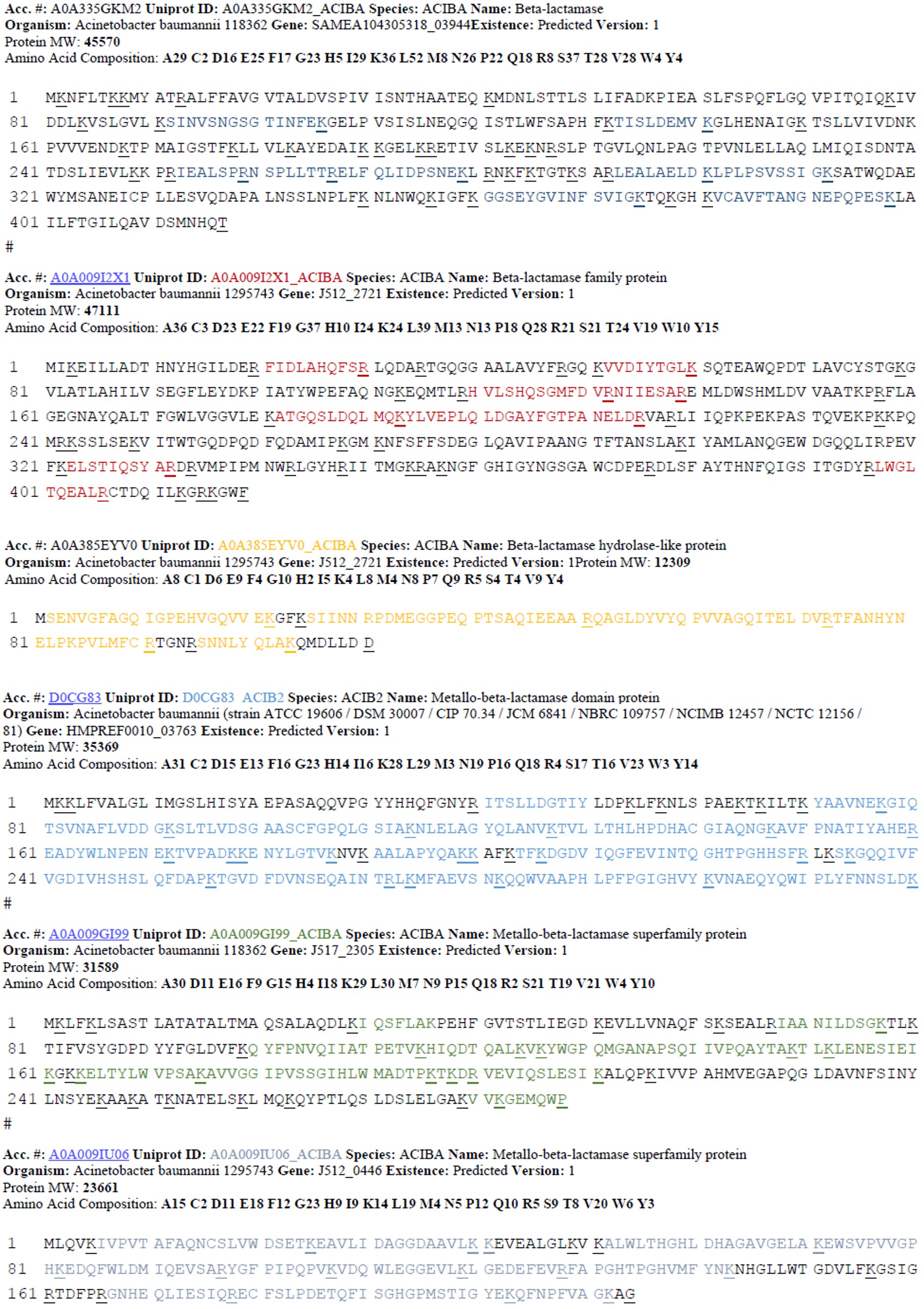


**
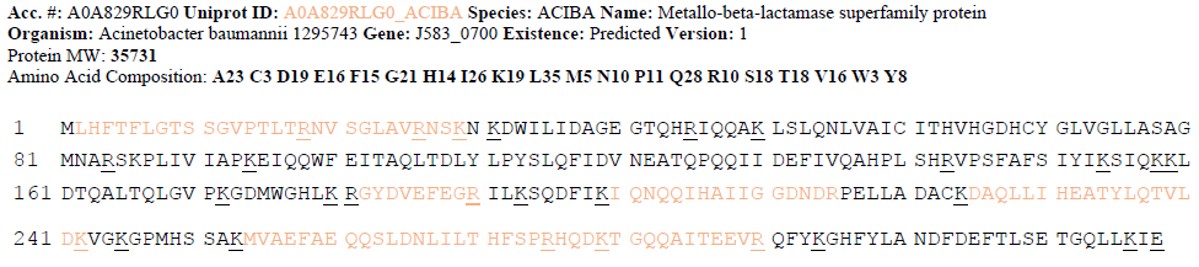
**


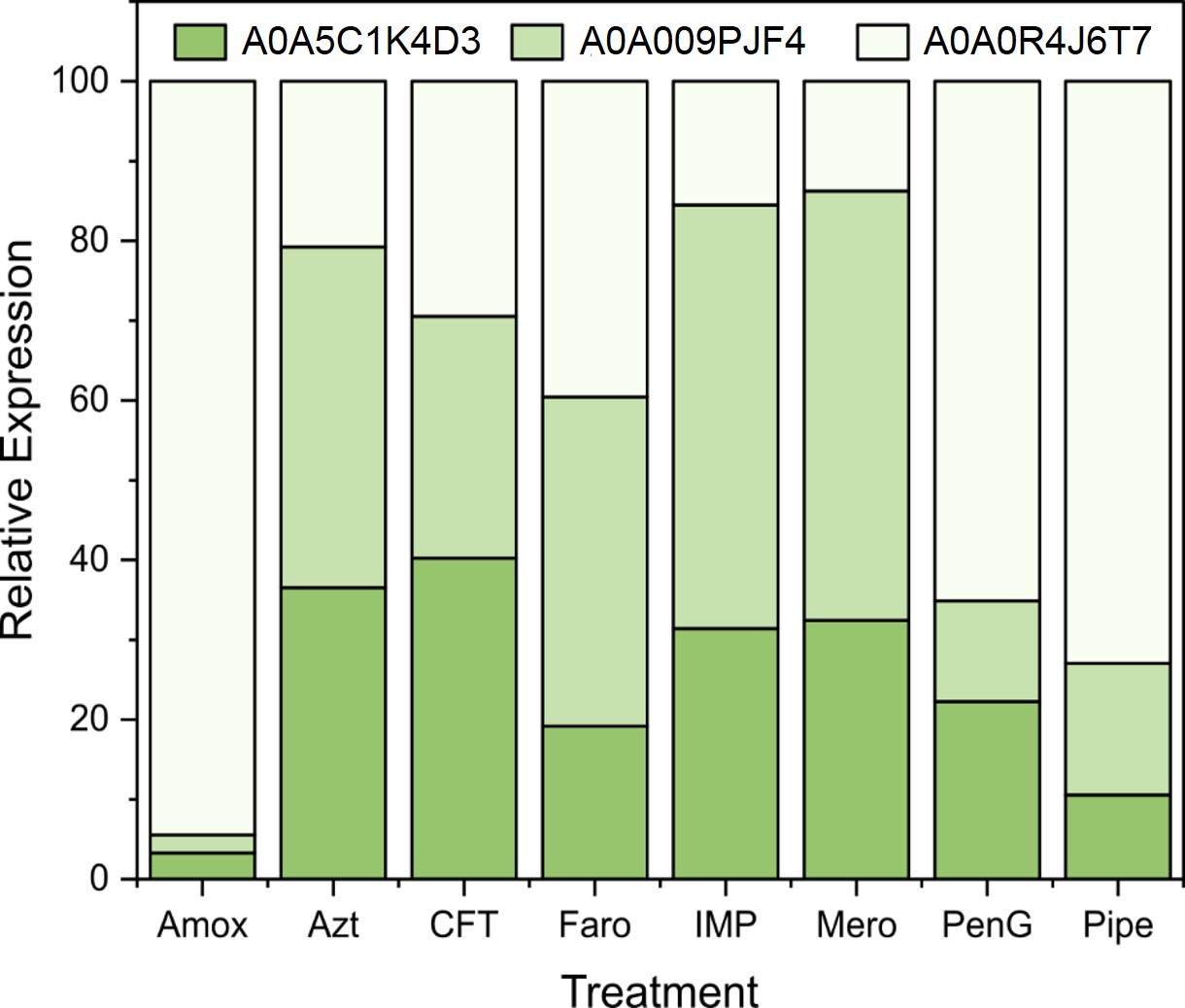


**Supplementary Figure S5.** Expanded comparison of relative Class C β-lactamase expression by Ab 19606 after treatment with various antibiotics.
